# Supplementary material for: Evaluating the Talbot-Plateau law
Source: Front Neurosci. 2023 Apr 27;17:1169162. doi: 10.3389/fnins.2023.1169162 (PMC10172486; doi:10.3389/fnins.2023.1169162)
Supplement: Supplementary file 1 [file Data_Sheet_1.docx]

Components of LED Display

The display consists of these major components:

- Host desktop computer: Apple Mac Mini. This runs experiment applications written in Tcl. Applications provide the experimenter user interface, setup control of treatments, trigger trials, and record results.
- Main display controller: mikroXMEGA embedded controller module on custom carrier board. This includes an Atmel ATXmega128A1 32MHz microcontroller serving as the global timing clock, multiple UARTs, I2C serial busses and digital outputs for communication with other components, a 12-bit DAC to set background LED intensity, and an ADC to measure voltage for self-calibration. Carrier board includes voltage follower buffers to distribute waveform (foreground) and background intensity signals to the 64 LED modules. The controller distributes display patterns to the LED modules, controls the waveform generator, and provides global timing for experiment trials.
- LED modules – 8x8 matrix of custom circuit boards. Each board includes an Atmel ATSAM3S1CA 64MHz controller, and drives an 8x8 matrix of LEDs with 64 parallel outputs. The outputs switch individual LEDs between foreground and background intensity control voltages, via a high-speed op-amp/transistor buffer circuit.
- Waveform Generator – HP33120A, 40Msample/s, 12-bit DAC + gain switching. Sample rate is 40M samples/sec, thus executing a sample in 25 ns.
- Power supply - Kepco 300W linear AC/DC supply

Key components on LED modules:

- OSRAM LS E65F LEDs: 633nm, 60 degree viewing angle, 4Cd at 50mA
- NXP NX3L4053 triple SPDT analog switch, 0.5ohm on resistance, 350mA capacity, switch time being 50 ns.
- MAXIM MAX4392 85MHz op amp. This has a bandwidth of 85MHz, response time being 12 ns.
- ZETEX ZXTN25012EZ NPN transistor, 6.5A, 70 ns rise/fall
