# Supplementary material for: Evaluating the Talbot-Plateau law
Source: Front Neurosci. 2023 Apr 27;17:1169162. doi: 10.3389/fnins.2023.1169162 (PMC10172486; doi:10.3389/fnins.2023.1169162)
Supplement: Supplementary file 2 [file Table_1.docx]

| **Letter** | **width/dots** | **width/arc°** | **height/dots** | **height/arc°** | **# dots** |
| --- | --- | --- | --- | --- | --- |
| **A** | **19** | **2.85** | **21** | **3.16** | **149** |
| **B** | **16** | **2.39** | **21** | **3.16** | **197** |
| **C** | **19** | **2.85** | **21** | **3.16** | **144** |
| **D** | **18** | **2.7** | **21** | **3.16** | **179** |
| **E** | **15** | **2.24** | **21** | **3.16** | **168** |
| **F** | **15** | **2.24** | **21** | **3.16** | **129** |
| **G** | **20** | **3** | **21** | **3.16** | **182** |
| **H** | **17** | **2.54** | **21** | **3.16** | **159** |
| **I** | **3** | **0.39** | **21** | **3.16** | **63** |
| **J** | **12** | **1.77** | **21** | **3.16** | **92** |
| **K** | **17** | **2.54** | **21** | **3.16** | **155** |
| **L** | **13** | **1.93** | **21** | **3.16** | **93** |
| **M** | **21** | **3.16** | **21** | **3.16** | **234** |
| **N** | **17** | **2.54** | **21** | **3.16** | **177** |
| **O** | **21** | **3.16** | **21** | **3.16** | **173** |
| **P** | **16** | **2.39** | **21** | **3.16** | **152** |
| **Q** | **21** | **3.16** | **22** | **3.31** | **190** |
| **R** | **18** | **2.7** | **21** | **3.16** | **190** |
| **S** | **16** | **2.39** | **21** | **3.16** | **153** |
| **T** | **17** | **2.54** | **21** | **3.16** | **105** |
| **U** | **17** | **2.54** | **21** | **3.16** | **145** |
| **V** | **19** | **2.85** | **21** | **3.16** | **129** |
| **W** | **29** | **4.39** | **21** | **3.16** | **244** |
| **X** | **19** | **2.85** | **21** | **3.16** | **138** |
| **Y** | **19** | **2.85** | **21** | **3.16** | **102** |
| **Z** | **17** | **2.54** | **21** | **3.16** | **152** |

**Supplemental Table 1.** The height and width of each letter is specified as dot count and as degrees of visual angle. The latter was measured from the outer edges of the most extreme dots. Two dots added to the tail of the Q produced an increase in height. Mean width of letters was 17.3 dots (2.60 arc°). Mean number of dots in the letter patterns was 153.6.
